# How Can Multi‐Criteria Portfolio Decision Analysis Help Mitigate Mis/Disinformation?

**DOI:** 10.1111/risa.70369

**Published:** 2026-09-28

**Authors:** Ahti Salo, Leevi Olander, Tuomas Raivio

**Affiliations:** ^1^ Systems Analysis Laboratory Department of Mathematics and Systems Analysis Aalto University School of Science Aalto Finland

**Keywords:** decision analysis, disinformation, governance, misinformation, multi‐criteria decision making, resilience

## Abstract

In this paper, we examine and illustrate the relevance of multi‐criteria portfolio decision analysis as an approach to addressing mis/disinformation risks. After summarizing representative applications of these methods, we draw on the framework for societal resilience developed in the Finnish COVID‐19 Science to Panel to elaborate on the range of capabilities that can be leveraged to mitigate mis/disinformation, for example, by maintaining alertness, implementing risk management actions, and interacting with stakeholders to promote awareness. We then present a numerical case study that illustrates how multi‐criteria portfolio decision analysis can be combined with probabilistic approaches for modeling voting behavior and the spread of mis/disinformation to identify cost‐efficient strategies consisting of combinations of risk management actions. We conclude by discussing some of the challenges posed by mis/disinformation.

## 1 Introduction

The creation, dissemination, adoption, and use of false information are growing concerns in modern societies. Rapid dissemination of information across media channels, particularly social media platforms, has become easier, while verifying claims has become more challenging due to limited transparency and access to trustworthy sources (Agarwal and Alsaeedi 2020). Whether the creation and dissemination of false information result from inadvertent errors (misinformation) or are deliberate, with the intent to cause harm (disinformation), their influence on subsequent decision‐making processes can pose substantial risks to individuals and societal structures (Aven and Thekdi 2022). Therefore, it is crucial to investigate potential risk management actions that can be implemented at various stages of the process to mitigate harmful consequences.

Finland has been reported to be more resilient to false and misleading content than most other countries, as indicated by its highest average truth quest score in a comprehensive survey of 21 countries (OECD 2024b; see also Saari et al. 2025). From this perspective, it is instructive to examine how the challenges posed by mis/disinformation are portrayed in the Report of the Future, published by the Finnish Government in September 2025 (Government of Finland 2025). This report is a forward‐looking strategic document that aims to prepare policymakers for potential societal, technological, environmental, and geopolitical changes, drawing on comprehensive coverage of domestic and international developments.

The most recent report (Government of Finland 2025) devotes considerable attention to mis/disinformation, which is seen as a primary driver of the decline in democracy and institutional trust. In particular, the report warns that AI systems can threaten institutional independence and undermine the rule of law. Rapid technological advancements can create an unpredictable landscape in which traditional democratic safeguards may fail to withstand sophisticated manipulation. The rise of disjointed “information bubbles” and “parallel realities” can make it increasingly difficult for opposing groups to understand one another, undermining social cohesion, fuelling social unrest, and weakening the factual basis of democratic discourse. The information environment is considered a central arena for geopolitical rivalry, where both state and non‐state actors manipulate information to advance their strategic goals.

Overall, the report portrays mis/disinformation as a systemic challenge that calls for coordinated policy responses across regulatory, educational, technological, and social sectors, with the aim of strengthening capabilities at multiple levels, spanning international, national, and regional organizations, as well as local communities and individual citizens. The shaping of an adequate risk management strategy against mis/disinformation therefore involves the development, selection, and implementation of actions that are jointly effective in countering harmful impacts. In practice, the preparation, selection, and implementation of such actions may have to satisfy various feasibility constraints related to the availability of human and financial resources, as well as technological capabilities.

An important distinction can be made between methods intended to support *preventive risk management* and those intended to select *reactive responses* to mis/disinformation in an ongoing crisis (cf. Boin et al. 2010). In the former, preventive setting, conditions for conducting extensive studies are better, as much more time and effort can be devoted to the analysis. Such studies can, for example, be conducted by synthesizing a broad range of documents to extract a systematically structured set of value‐focused objectives that can then be leveraged to support subsequent decision‐making (see, e.g., Simon et al. 2014). Conversely, during an ongoing crisis, pressure to react quickly is likely to limit the breadth (scope of topics) and depth (level of detail in exploring these topics) of the analysis. This notwithstanding, it may be possible to lay a stronger foundation for reactive risk management through structured decision processes that are rehearsed in advance with decision‐makers and, where appropriate, external stakeholders. In defining such processes, one can draw on experiences from other contexts, such as selecting remediation options in nuclear risk management (see, e.g., Geldermann et al. 2009).

In the context of mis/disinformation, multi‐criteria methods, most notably multi‐criteria decision analysis (MCDA) (see, e.g., French 1993; Greco et al. 2016), are helpful because they enable the systematic evaluation of risk management actions, taking into account the various objectives involved in developing a sound risk management plan and decision‐makers' preferences for achieving these objectives. These objectives can encompass a broad range of impacts, such as limiting the size of population segments reached by mis/disinformation or mitigating its effects on their behavior. The range of alternative actions can be equally wide. For example, these actions can seek to enhance technical capabilities for detecting mis/disinformation, impose tighter regulatory restrictions, or raise awareness through targeted educational campaigns.

In general, the formulation of alternative actions from which risk management strategies are built can be guided by frameworks for societal resilience. The dimensions of such frameworks can represent, for example, specific sectors in which actions are applied (e.g., health care, finance, transportation) or different phases of the risk management process (e.g., identification, implementation, monitoring). Conceptually and operationally, these frameworks can serve as checklists to ensure that the selected risk management actions adequately cover these dimensions (see, e.g., Rød et al. 2025 and references therein), recognizing that best strategies typically involve combinations (portfolios) of actions that must be implemented together to ensure effectiveness. For example, the ability to identify mis/disinformation does not suffice unless accompanied by actions that limit its impacts.

As a result, methods of multi‐criteria portfolio decision analysis (see, e.g., Salo et al. 2011; Liesiö et al. 2021) can help assess the performance of different strategies with respect to relevant objectives, such as minimizing the extent to which mis/disinformation propagates or affects those exposed to it. Specifically, these methods make it possible to determine which strategies, defined as portfolios of actions, offer the most value in supporting the attainment of these objectives, subject to relevant resource) and other constraints that apply. This can be achieved by constructing a multi‐criteria model that, on the one hand, captures decision‐makers' preferences for criteria representing the attainment of multiple objectives and, on the other hand, aggregates the evaluations of strategies with regard to these criteria into an overall measure of value, thereby identifying the strategy for which this value is highest.

Against the above background, the methodological contribution of this paper lies in proposing a systemic approach that combines multi‐criteria portfolio decision analysis with models describing the uncertain propagation of mis/disinformation in networks of information users who may be affected by or spread such information. Our case study highlights the need for such an approach, as the impacts of risk management actions often depend on what other actions are implemented. Specifically, the impact interactions among the actions are captured by the propagation model, while the attainment of overall risk‐mitigation objectives (such as minimizing exposure to mis/disinformation) is assessed with the multi‐criteria model. Furthermore, we relate different types of risk management actions to a capability framework for societal resilience, noting that using this framework as a checklist can stimulate the development of additional risk management actions.

Because the proposed approach helps identify cost‐efficient risk management strategies to counter mis/disinformation, it can guide investment planning. It also allows these strategies to be optimized under different scenarios that reflect alternative assumptions about the future. These two uses constitute contributions to the future research directions outlined by Hunt et al. (2020).

The rest of this paper is organized as follows. Section 2 briefly summarizes the key characteristics of multi‐criteria methods, and Section 3 reviews selected case studies in which these methods have been used to counter mis/disinformation. Drawing on the work of the Finnish COVID‐19 Science Panel, Section 4 outlines a capability framework for promoting societal resilience and interprets it in the context of mis/disinformation. Section 5 presents a case study showing how multi‐criteria portfolio decision analysis can be combined with probabilistic models of disinformation propagation to formulate cost‐efficient risk‐mitigation strategies. Section 6 discusses possible extensions of the proposed approach, and Section 7 concludes.

## 2 Multi‐Criteria Methods

In the literature and professional practice, there is a wide variety of multi‐criteria methods (see, e.g., Greco et al. 2016), which differ in their theoretical foundations and in the procedural steps for eliciting and synthesizing preference information. As a rule, one key rationale for using multi‐criteria methods is to improve the quality of decision‐making, for example, by promoting adherence to reasonable axioms of rational decision‐making, such as the independence of irrelevant alternatives (Keeney and Raiffa 1976; French 1993). Against this background, we advocate sound methods that adhere to widely embraced axioms of rationality.

This is the case with multi‐attribute value theory (MAVT; see, e.g., Belton 1998; French 1993), which is based on the explicit articulation of the values (e.g., ensuring safety) that decision‐makers seek to realize and can therefore be viewed as objectives. Specifically, the attainment of each objective is assessed by specifying a corresponding criterion, which is then used as an attribute to measure the performance of all alternatives. Mathematically, the structure of MAVT models is given in its simplest form by the additive sum

(1) $$V\left(x\right)=\sum_{i=1}^{n}w_{i\;}v_{i}\left(x_{i}\right),$$

where V(x) is the overall value of alternative $x=(x_{1},\text{…},\;x_{n})$, characterized by its performance levels $x_{i}$ on each of the *n* criteria; $w_{i}$ is the criterion weight associated with the *i*th criterion; and $v_{i}\left(x_{i}\right)$ is the score that is attached to the performance level $x_{i}$ by employing a single‐attribute value function $v_{i}$(.) from the domain of performance levels to nonnegative real numbers. The scores are usually normalized to the interval [0,1], following the convention that the score for the worst performance level is 0 and that for the best performance level is 1.

By convention, the weights are $w_{i}$ positive and are usually normalized to sum to one, that is, $\sum_{i=1}^{n}w_{i}=1$. Thus, given the additive structure of Equation (1), the weight $w_{i}$ represents the additional contribution to the overall value that can be gained by improving the performance on criterion *i* from its worst level to the best one. In particular, this weight is not an absolute measure of importance but reflects how much additional value is attained by improving performance on this criterion relative to the improvements that can be made on other criteria (Keeney and Raiffa 1976). Moreover, the additive value function in Equation (1) is compensatory in the sense that weaker performance on some criterion can be compensated through improved performance on other criteria.

Once the additive value function in (1) has been built and its numerical parameters, most notably alternatives' scores $v_{i}$($x_{i}$) and criteria weights $w_{i}$, have been elicited, the corresponding overall values V(x) can be used to inform decisions by recommending the alternative with the highest overall value. The process of eliciting these parameters, for instance, through structured interviews or facilitated workshops, encourages respondents to articulate how the alternatives contribute to the objectives represented by the criteria. This process can offer insights even if Equation (1) is not formally employed to prioritize alternatives. As a result, we do not view multi‐criteria methods merely as an approach to produce explicit recommendations about choices among alternatives. Rather, we consider multi‐criteria methods as enablers of systematically structured processes in which alternatives are explicitly defined and evaluated against criteria that represent stated objectives, without requiring that these evaluations be necessarily combined to yield a strict ranking of alternatives.

In general, offering a structured framework for systematically evaluating alternative actions provides process benefits by creating a traceable, transparent record that can be used to justify the chosen actions and revisited later in light of new evidence (Geldermann et al. 2009). This can be a notable benefit that promotes trust and can therefore enhance the positive impact of the formal decision process on the decision outcome. Further benefits (for an overview, see Salo et al. 2021) include the possibility of reaching decisions more quickly, for example, by developing and applying suitable decision templates. Consistency can be enhanced through the systematic consideration of all alternatives, while collective learning is supported by a constructive dialog among the participants.

## 3 Applications of Multi‐Criteria Methods in Countering Mis/Disinformation

We next summarize selected applications of multi‐criteria methods in the context of mis/disinformation. As our selection is necessarily limited in scope, we refer to comprehensive reviews (e.g., de Almeida et al. 2017; Maček et al. 2020).

- Komendantova et al. (2021) propose a decision‐analytic framework to evaluate tools and systems for combating misinformation on social media. They present a multi‐criteria approach in which preferences are elicited from stakeholders, including policymakers, journalists, and citizens. Based on the experiences using this approach in Austria, Greece, and Sweden, they note that some of the most valued features of misinformation tools include the ability to study the accountability of distributors of false content, the lifecycle and pathways of misinformation spread, and the perceived intention to deceive. The experiences also suggest that cultural and stakeholder differences shape preferences for tools and should thus be accounted for in countering misinformation.
- To support the detection of fake news, Abrar et al. (2023) conducted a comprehensive Multivocal Literature Review based on 161 published and 61 unpublished studies. They identified 14 credibility factors, such as “reporter reputation” and “impartiality,” which were then analyzed using statistical tests and the analytic hierarchy process (AHP) to determine the most important factors across different domains.
- Bajpai and Chaturvedi (2024) propose an integrated approach that combines MCDA with epidemic diffusion modeling to prevent the early spread of rumors in social networks. They motivate their approach by noting the need to (1) debunk rumors quickly, (2) reduce delays by initiating the diffusion of counter‐rumors promptly, and (3) select trusted nodes to spread counter‐rumors. The identification of influential nodes is supported by a variant of the AHP, while the diffusion of rumors is modeled by extending the susceptible–infected–recovered (SIR) epidemic model. Illustrative examples are provided using six real‐world social network datasets.
- Luo et al. (2023) examine the reputational and economic losses companies may incur from the spread of negative online content in complex public‐opinion environments. They present an extensive framework that combines (i) a multi‐criteria approach to determine indicator weights for detecting such content; (ii) probabilistic hesitant fuzzy sets to account for uncertainties in experts' judgments about these weights (see also Viviani and Pasi 2017); and (iii) a compromise solution method to evaluate possible pathways for information spread. They demonstrate the framework by prioritizing information pathways in a case study from the catering industry but do not explicitly address how to handle these pathways.
- da Costa et al. (2025) design a structured decision‐making framework for evaluating and choosing countermeasures against fake news and disinformation. Specifically, they view fake news as a form of digital risk to be addressed through interventions whose attractiveness and effectiveness enforcement and policy actors can assess. The framework systematically considers multiple criteria, such as costs and impact on risk reduction, when developing strategies in response to high‐impact disinformation events. In particular, effective mitigation requires adequate risk prioritization and targeted resource allocation.
- Ganin et al. (2020) note that tools for assessing cybersecurity risks often fail to fully incorporate the three core components (threats, vulnerabilities, and consequences) that need to be considered when managing risks across the physical, informational, and social domains of cyber systems. Motivated by this insight, they propose an MCDA model to quantify these three components and compare cybersecurity management strategies. They also describe a hypothetical case study on evaluating five different countermeasures. The model offers a transparent, value‐based process that aligns chosen strategies with stakeholders' preferences.
- Nielsen et al. (2019) examine the implications of potential “fake” information in decision analysis, emphasizing the need to treat all information, whether fake or real, using the same Bayesian decision analysis approach. Specifically, they propose a decision‐analytic approach that explicates multiple system models. Their framework helps assess the value of obtaining better information, for example, by removing false data. This can provide useful insights, even if the pressing realities of ongoing crises may constrain the ability to explicate and validate such system models.

To assess the potential for managing mis/disinformation risks, it is instructive to examine realized risks with similar characteristics. In this regard, the COVID‐19 pandemic is relevant, as false claims about vaccine safety were a major concern for healthcare leaders, and individual choices to refuse vaccination posed significant risks to the population (see, e.g., Vujovich‐Dunn et al. 2021; Lazarus et al. 2022). Furthermore, in both cases—pathogens and mis/disinformation—the processes leading to harmful consequences have been analyzed using similar concepts, such as contagiousness, susceptibility, infection, and recovery (cf. Jamalzadeh et al. 2022). Even the notion of an incubation period, which has a clear meaning in epidemiology, can be interpreted as the delay between the spread of mis/disinformation and its impact on behavior (see, e.g., Lv et al. 2022).

Against this background, Dillon et al. (2023) offer an enlightening review of how decision analysis can guide decision‐makers during an ongoing pandemic by characterizing key uncertainties, eliciting decision‐makers' preferences, and systematically evaluating alternative courses of action. However, they focus primarily on individual decisions, providing case‐by‐case guidance for each decision. This is useful for preparing decision templates that can be documented in advance and used during training to improve the consistency and comprehensiveness of decisions regarding alternative risk management actions in an eventual crisis.

Nonetheless, focusing on individual actions has limitations for addressing the complex relationships among them because it does not recognize that the strategy to be implemented will most likely comprise many actions. In particular, in an ongoing crisis, the de facto strategy can be viewed as the realization of interconnected decisions made across various levels of administration, under significant time pressure and based on incomplete, ambiguous, and highly contested information. Furthermore, in a crisis, the pressure to act can be overwhelming, leaving less time and resources for detailed analysis. From this perspective, it is instructive to consider how risk management actions can be analyzed jointly with portfolio decision analysis against the backdrop of general frameworks of societal resilience that draw attention to interdependencies among risk management actions and the ensuing need for adequate coordination among them (see, e.g., Agarwal and Alsaeedi 2020; Raetze et al. 2021; Westover 2024).

## 4 A Capability Framework for Societal Resilience

The concept of resilience, and more specifically societal and social resilience, has been examined from complementary perspectives that span a range of capacities needed in the face of disruptions. Keck and Sakdapolrak (2013), for example, distinguish between coping, adaptive, and transformative capacities, which, respectively, refer to the abilities to overcome adversity, to learn from past experiences, and to adjust to future challenges through institutions that foster sustainable robustness. Lorenz (2013) pinpoints the need for participative capacity, which refers to the system's ability to change its own structure in response to interventions by other systems. Lucini (2014) highlights the capacities, abilities, and competencies needed to cope with stress, a crisis, or a disaster and to bounce back to preexisting conditions of life. Moya and Goenechea (2022) maintain that social resilience must be conceived as a dynamic, multilevel, and evolutionary process to help societies adapt and transform themselves. In general, these perspectives share many similarities: they recognize the need to absorb and learn from shocks and to pursue transformative change to be better prepared for future crises in specific contexts. For example, Hochrainer‐Stigler et al. (2020) present a standardized community resilience framework tailored to measuring disruptions due to flooding.

It is instructive to examine mis/disinformation risks against the backdrop of resilience frameworks that help societies prepare for, endure, and recover from them. For example, Nasery et al. (2023) adapt the Security Action Cycle model (Straub and Welke 1998) to the context of fake news on social media by distinguishing the sequential phases of *deterrence*, *prevention*, *detection*, and *mitigation/remedy*. Building on an extensive literature review, they discuss approaches to address the risks of fake news across these four phases, elaborating on strategies, relevant actors, their roles, and research gaps for each phase. They argue that because of the complexities of how fake news spreads, effective countermeasures need to span multiple disciplines and stakeholder groups. They also stress the need for integrated tools and longitudinal studies to facilitate a shift from isolated tactics to a more systemic, process‐oriented approach to conducting analytical studies, which is one of the motivations of the present paper (see also Bondielli and Marcelloni 2019; Zhang et al. 2019; Westover 2024).

Rød et al. (2025) provide a comprehensive literature review of research published from 2018 to 2022 on disinformation in highly digitized societies, in which state infrastructure and civil society use integrated digital technologies in daily governance and civic engagement. Many (but not all) of these societies can be viewed as digitally mature democracies thanks to governing institutions that promote regulatory frameworks to protect democratic values from digital threats (see, e.g., OECD 2024a). Rød et al. also propose a structured framework that categorizes counter‐disinformation measures across multiple dimensions, including *legal/regulatory*, *educational*, *political/governance*, *psychological/social–psychological*, and *technological* factors. This framework can serve as a practical tool to evaluate the maturity of counter‐disinformation efforts and, more specifically, to help stakeholders benchmark and develop strategies for improved resilience.

These two frameworks are complementary. The framework in Nasery et al. (2023) has a strong temporal “process” focus on the operational management of risks related to false news in the four different phases. In contrast, the framework of Rød et al. (2025) adopts a more static, “vertical” approach in which the preconditions of and contributors to societal resilience are itemized separately within each sectoral dimension.

As a complement to these two frameworks, we next outline the capability framework for societal resilience developed by Finland's COVID‐19 Science Panel. This framework differs from those above in its emphasis on *capabilities* (rather than phases or sectors in society) that may be needed to navigate dynamic phenomena characterized by interactions between communities, social contagion, significant uncertainties, and interdependent decision options. Although originally motivated by the acute need to manage the COVID‐19 pandemic, this framework is generic in providing a transferable tool for examining and structuring capabilities that strengthen resilience by helping societies anticipate, absorb, and recover from disruptions. Here, for the purposes of this paper, capabilities are understood as the *planning and execution of specific risk management actions that contribute toward these aims* (for a review of related definitions of capabilities, see Lindbom et al. 2015).

Administratively, the COVID‐19 Science panel was appointed by the Prime Minister's Office in April 2020 to support the Finnish Government in managing the pandemic. It consisted of 13 leading researchers from different fields^1^ who were asked to synthesize information to support recovery planning by offering recommendations in its 115‐page main report, aimed primarily at the government but also at many other institutional actors and stakeholders, including individual citizens (Government of Finland 2020).

Specifically, this framework (see Figure 1) features four dimensions, referred to as capability pillars (see also Kontogiannis et al. 2017), which represent the different kinds of capabilities that are needed to prepare for and navigate societal crises even in the presence of high stakes, limited information, pressing schedules, and major uncertainties about the effectiveness of countermeasures and their broader consequences:

1. *Maintain forward‐looking alertness* by establishing a timely, comprehensive foundation consisting of validated information for decision‐making.
2. *Enact decision‐making processes* that build on this foundation to help decision‐makers prepare and choose among alternative risk management actions based on a coherent, systematic evaluation, which may involve stakeholder consultations.
3. *Implement risk management actions* effectively to support the attainment of objectives for chosen actions.
4. *Interact with stakeholders* through purposeful, continual, participatory, and multidirectional communication efforts that build trust, shape risk perceptions, and aid the successful implementation of risk management actions, such as encouraging voluntary collaboration among stakeholders.

**FIGURE 1:**
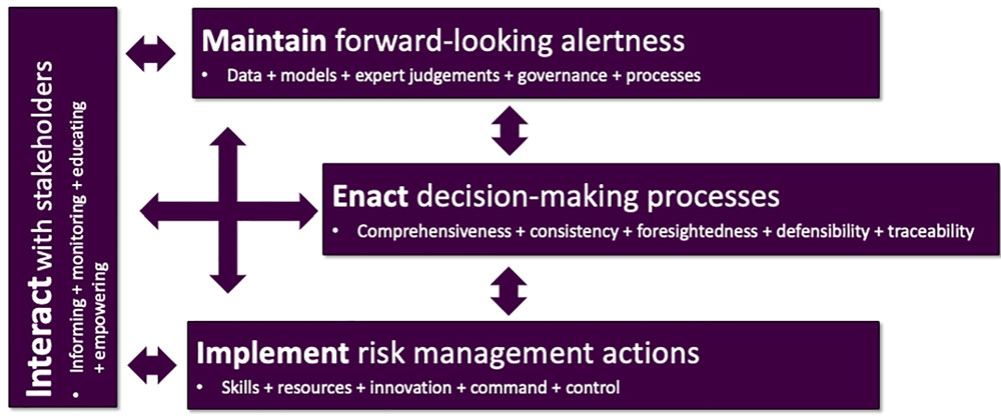
The four pillars in the capability framework for societal resilience.

We next elaborate on these dimensions in the context of mis/disinformation.

I Maintain forward‐looking alertness

This first capability pillar refers, in a broad sense, to the ability to build and maintain required information resources for building up‐to‐date, balanced, comprehensive, and forward‐looking situational awareness and alertness as a basis for decision‐making. It includes data resources and analytical tools to help detect mis/disinformation, but it also covers other sources of information, such as solicitation of expert judgments and even laypeople's opinions on the susceptibility of specific population segments to targeted disinformation campaigns. Because timeliness is a key concern, there may be a need to conduct continuous horizon scanning to monitor the environment and identify relevant trends, observations, or hypotheses about mis/disinformation that, after validation, can be added to the approved pool of information resources.

The identification of weak signals (see Hines et al. 2019 for a literature review) can be supported by a systematic multi‐criteria assessment based on criteria such as relevance, novelty, and probability of occurrence (see, e.g., Könnölä et al. 2012). Concretely, building on the recognition that weak signals are likely to be those that are given a high score on such criteria by some but not all respondents, the computation of dispersion metrics such as the variance of the respondents' scores on the criteria can be used as an indicator of whether a proposed signal could be viewed as a weak one. For a detailed case study on such an approach, applied to the generation of innovation ideas, see Könnölä et al. (2007).

II Enact decision‐making processes

The second capability pillar consists of the capabilities required to integrate relevant information resources into structured decision‐making processes. Apart from covering the characteristics of the specific occurrence of mis/disinformation and its potential impacts, these resources need to include information about the risk management actions currently available or those that can be developed. The latter perspective is vital, as focusing only on mis/disinformation, without links to actions that may be taken to curtail it, will not limit harmful consequences.

By design, multi‐criteria methods strengthen this capability pillar by helping make choices among available courses of action (cf. Komendantova et al. 2021). When selecting actions, constraints often arise, for example, from the availability of scarce resources or specialized expertise. Such constraints affect the feasibility of selected actions, which may compete for resources from the same pool. Here, methods of portfolio decision analysis (see, e.g., Salo et al. 2011; Liesiö et al. 2021) can be applied to select which actions to implement jointly to build a portfolio that performs as well as possible with respect to relevant criteria, subject to resource availability and other constraints. Such analyses can also be used to identify what resources would be needed to attain performance targets or what kinds of new actions, enabled by technological advances, for instance, would complement those actions that have been available earlier.

III Implement risk management actions

This capability pillar refers to the operational implementation of the selected actions as planned. However, as the effectiveness of the actions may involve uncertainties, it is important to monitor the targeted effectiveness as well as possible impacts more generally, and if necessary, to make adjustments “along the way” (e.g., improving measures that aim to inoculate the population against mis/disinformation; see Bessarabova et al. 2024), rather than choosing irreversible strategies that could have unintended effects (such as denying all access to social media platforms, which could lead to widespread resentment and mistrust of authorities).

IV Interact with stakeholders

This fourth capability pillar links to those above. It is a vital part of (i) raising broader societal awareness of mis/disinformation, which is ultimately influenced by interactions among different stakeholder groups in society; (ii) identifying effective risk management actions by involving stakeholders who often have a better “grass‐root” level understanding of information sharing patterns than administrators and policymakers; and (iii) enabling commitment and successful implementation of these actions through collaboration among key societal stakeholders (e.g., willingness to alert peers about false statements within one's social networks).

Interactions related to this capability pillar should not be viewed as one‐way, based on the idea that “correct” information comes from and is shared by policymakers and administrators. Instead, when considered alongside the other three capability pillars, these interactions can more fruitfully be understood as ongoing, multidirectional, participatory activities that span local communities and social networks across different time horizons to enhance resilience to mis/disinformation, for example, by fostering cohesion and strengthening adaptive and transformative capacities ex ante as well as coping capabilities ex post (see, e.g., Aldrich and Meyer 2014; Keck and Sakdapolrak 2013; Lorenz 2013; Townshend et al. 2015). Furthermore, because mis/disinformation can cause greater harm when believed and acted upon, activities that focus on informing, educating, and empowering stakeholder groups about the risks of mis/disinformation are essential (Carmi et al. 2020). In low‐trust societies, the successful implementation of such activities may pose unique challenges, as administrative initiatives are often viewed with greater suspicion (see, e.g., Löfstedt 2005; Lenton et al. 2022).

### 4.1 The Systemic View

Because the four capability pillars represent distinct capabilities that can be manifested through the execution of different kinds of actions, part of the value of this framework lies in examining them together holistically. This can be especially important in organizations where the processes of information gathering, decision‐making, implementation, and communication span across administrative silos, as is often the case in public administrations.

In this regard, the capability framework can be harnessed as a checklist to ensure that all capability pillars receive due attention. It can also serve as a basis for a structured assessment process, in which possible actions associated with the different capability pillars are first elaborated and then evaluated against the aims of respective pillars to derive indicators that reflect the maturity of risk management capabilities. Such an evaluation can be carried out using measurement scales as in multi‐criteria methods (e.g., see Yoshiura et al. 2023).

This notwithstanding, the above capability framework should not be viewed as an MCDA model that represents objectives in the same way as the additive model in Equation (1). By construction, this additive model assumes that low performance on a criterion reflecting one objective can be compensated for by higher performance on another criterion. In contrast, this assumption of additivity does not hold when evaluating capabilities. To see this concretely, if the capabilities to prevent the spread of mis/disinformation are entirely lacking, this deficiency cannot be compensated for by improved capabilities for maintaining alertness. Conversely, alertness alone does not suffice unless accompanied by effective means to counter mis/disinformation through appropriate actions.

Accordingly, the capability framework in Figure 1 should more aptly be seen as a tool for multi‐criteria mapping, which is a reflective and participatory approach for systematically addressing complex problems from multiple perspectives with the aim of developing an improved shared understanding (see, e.g., Kuipers‐Dirven et al. 2023; Stirling and Coburn 2006). Compared with MAVT models, multi‐criteria mapping is less normative, as its benefits often arise from a structured dialog about the range of policy options rather than from clear‐cut decision recommendations (White 2017). This was also the case in the COVID‐19 Science Panel, as this framework catalyzed constructive discussions during joint meetings between the Panel and the Chiefs of Staff from different ministries.

Many of the responses to mis/disinformation in the Government's Report on the Future (Government of Finland 2025) can be examined in light of the capability framework in Figure 1. For example, responses that enhance citizens' skills in identifying mis/disinformation contribute to heightened awareness (Capability Pillar I); the development of proactive regulation is an enabler of timely decision‐making processes (Capability Pillar II); enhanced data protection measures and improved technical capabilities support the implementation of effective risk management actions (Capability Pillar III); and stakeholder interactions that promote media literacy and uphold the value of scientific thinking and research‐based information lessen the impacts of mis/disinformation among recipients (Capability Pillar IV).

Against this backdrop, Figure 2 shows how the proposed approach of multi‐criteria portfolio decision analysis builds on this framework. Here, the labels attached to the arrows characterize relationships between adjacent elements: for instance, capabilities enable the execution of risk management actions. This figure clearly distinguishes individual risk management actions from strategies because strategies typically consist of several actions. It also highlights that multi‐criteria evaluation should focus on the consequences associated with strategies (instead of individual actions, because these may exhibit strong interactions). In predicting these consequences, computational models representing the emergence, propagation, and impact of mis/disinformation can be helpful. For example, they make it possible to assess the benefits of introducing the possibility to implement new risk management actions in addition to those that are already available, as illustrated by the following case study.

**FIGURE 2:**
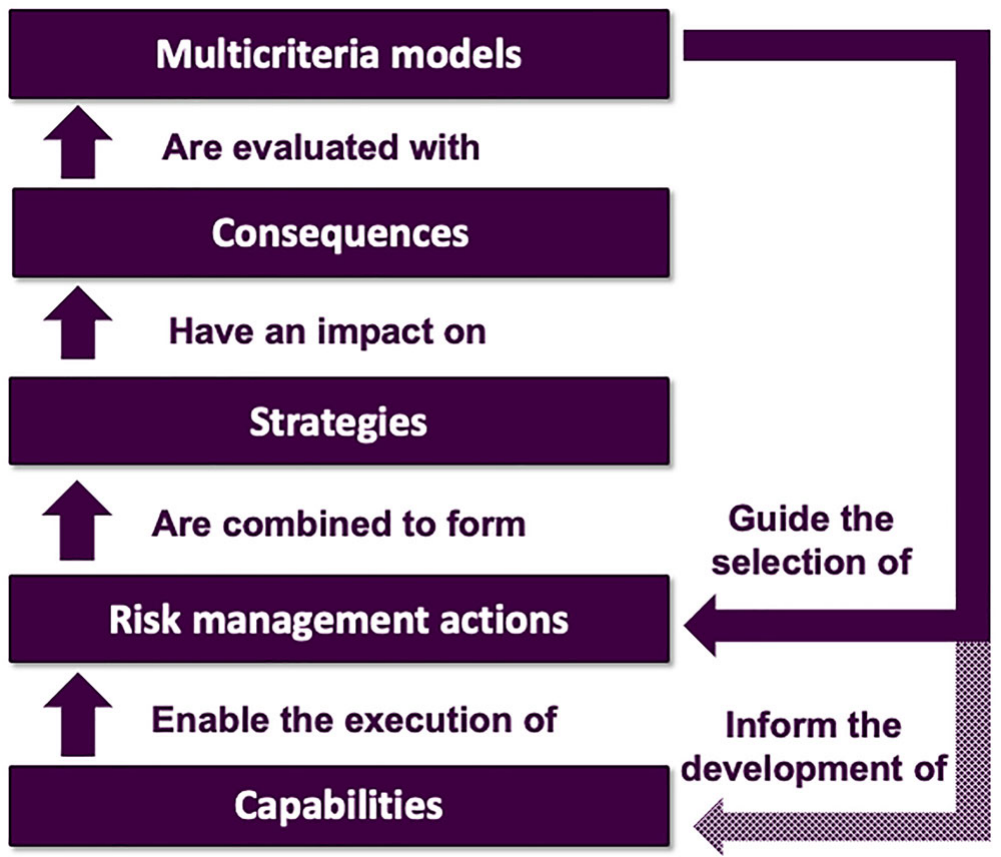
The main elements of the proposed multi‐criteria approach to evaluating strategies consisting of risk management actions.

## 5 A Case Study on Mitigating the Impact of Disinformation on Voting

The following case study illustrates how multi‐criteria portfolio decision analysis can be integrated with other analytical techniques, such as Bayesian modeling of voting behavior and cascade models for describing its propagation. This study, downsized and streamlined for transparency, builds partly on a real‐world scenario analysis engagement^2^ conducted in collaboration with authorities in preparation for national parliamentary elections.

Consider a scenario in which a municipal council consisting of 17 voters decides by majority vote whether to grant a permit for the construction of a contested production facility. At the outset, slightly more support exists for granting this permit. However, disinformation posted on one of three social media platforms can undermine this support by propagating to voters through the connections in Figure 3 (cf. Schneider et al. 2013). Before the vote, one platform, each with a one‐third probability, will be targeted with disinformation containing false claims that speak against granting the permit.

**FIGURE 3:**
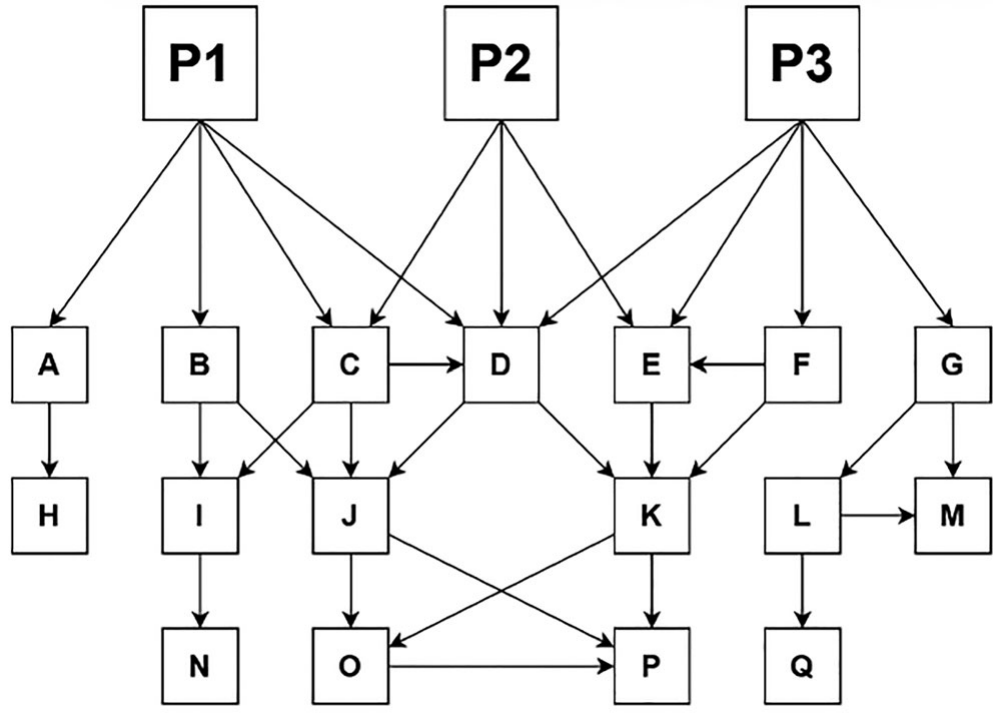
Network of platforms (large nodes at the top), voters (small nodes), and communication connections among platforms and voters (directed arrows).

The propagation of disinformation in Figure 3 is modeled by the independent cascade model (see, e.g., Wang et al. 2012; see Appendix A for details). If enough voters receive it and are influenced by it, the council's decision may be tipped toward permit denial. To counter the possibility of this inadvertent shift, there are three types of risk management actions:

1. *Monitoring*: Each platform can be monitored to detect disinformation.
2. *Isolation*: The attacked platform can be isolated to prevent the propagation of disinformation from it.
3. *Education*: Voters can be informed through an education program that reduces the impact of disinformation on voting behavior.

We assume that actions to monitor platforms and educate voters can be implemented at two levels of effectiveness. For monitoring, the less effective level costs 1 unit, and the more effective level costs 2 units. For education, the costs for the two levels are 2 and 4, respectively. Even if a platform is monitored and attacked, disinformation may go undetected, but there are no false indications of disinformation if the platform has not been attacked. In response to a positive monitoring indication, the attacked platform can be isolated for a cost of 8 units, payable up front. Thus, the costs arising from the choice of actions are not uncertain.

Note that the three types of risk management actions above can be related to the pillars of the capability framework in Figure 1. Specifically, monitoring attacks on the platforms helps *maintain enhanced awareness*; isolating platforms is a means to *implement risk management actions*; both variants of educating voters call for the capability to *interact with stakeholders*; and combining these elements to prepare a systemic analysis with ensuing recommendations helps *enact decision‐making processes*.

Specifically, voting behavior follows the beta distribution (see, e.g., Rigdon et al. 2009) in Figure 4 so that the impact of education is modeled by adjusting likelihood ratios (see Appendix A). The prior distribution of voters' initial probabilities, shown in blue, implies that the permit is granted with a probability of 0.572. The three other distributions, indicated by dashed lines, show the distributions for disinformed voters who have received either no, limited, or substantial education. Note that the distribution for more educated voters deviates less from the initial blue distribution.

**FIGURE 4:**
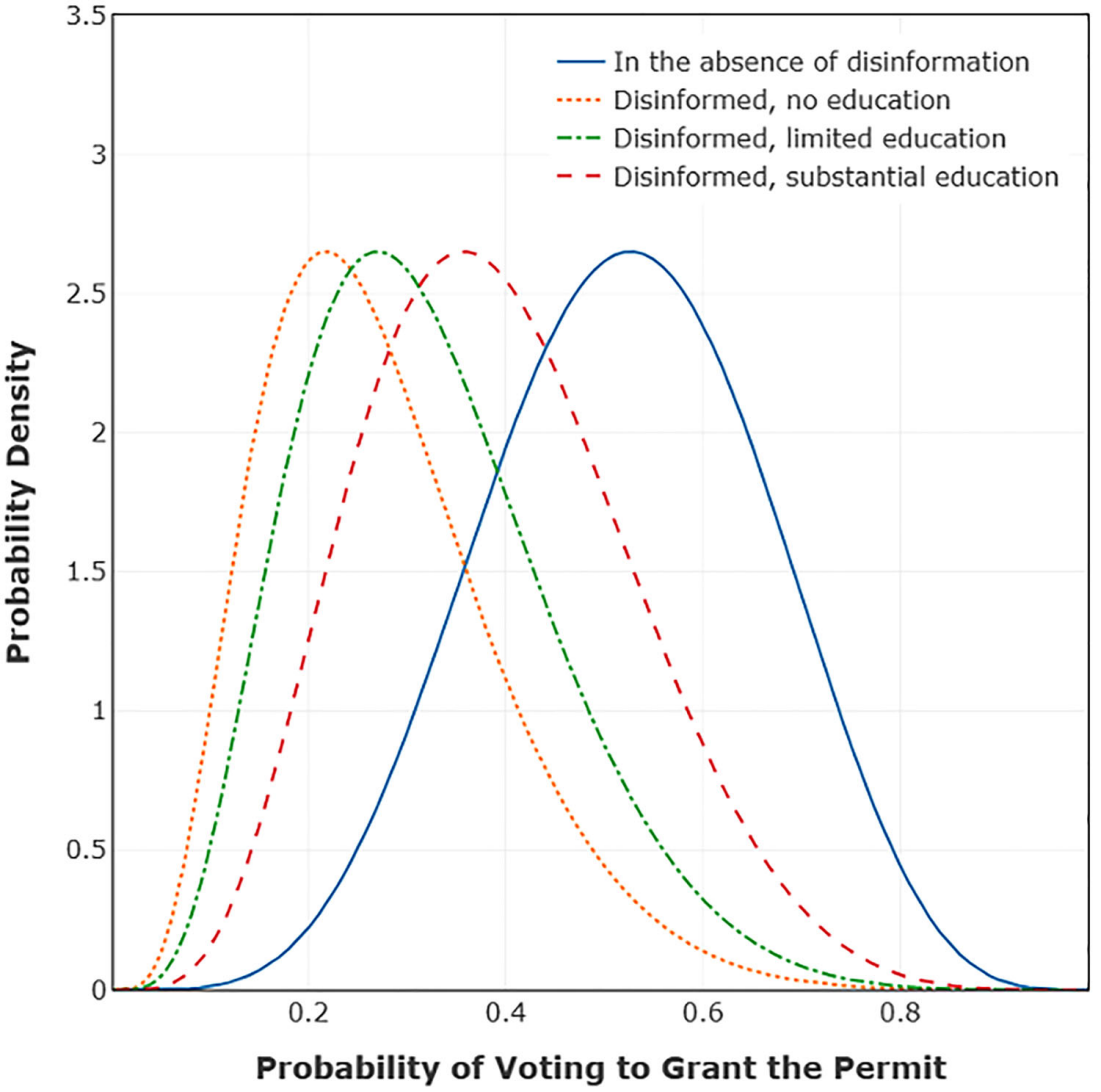
Beta‐distributed voting behavior based on disinformation and education status.

The three main objectives in this scenario are as follows:

(i) minimize the costs incurred due to the implementation of actions,
(ii) minimize the probability of disinformed voters (meaning that at least one voter is exposed to disinformation), and
(iii) minimize the impact of disinformation by lowering the probability that the permit is denied as a result of it.

In this example, there are 27 × 2 × 3 = 162 different strategies, each a portfolio of several risk management actions. Specifically, there are 3 platforms with 3 monitoring possibilities for each (i.e., no monitoring = 0, light monitoring = 1, close monitoring = 2), which gives 3 × 3 × 3 = 27 options; the action to isolate the attacked platform is binary, which gives 2 options; and finally, there are 3 options in providing education (i.e., no = 0, limited = 1, or substantial education = 2).

Of these 162 strategies, it is of interest to identify those that are Pareto‐optimal, in the sense that there is no other strategy that would perform as well on all objectives and, in addition, be strictly better on at least one of them.

To this end, we ran Monte Carlo simulations for each strategy to estimate its expected consequences with respect to the three objectives; see Figures 5 and 6. Out of the 162 strategies, only 11 are Pareto‐optimal in keeping with the above definition.

**FIGURE 5:**
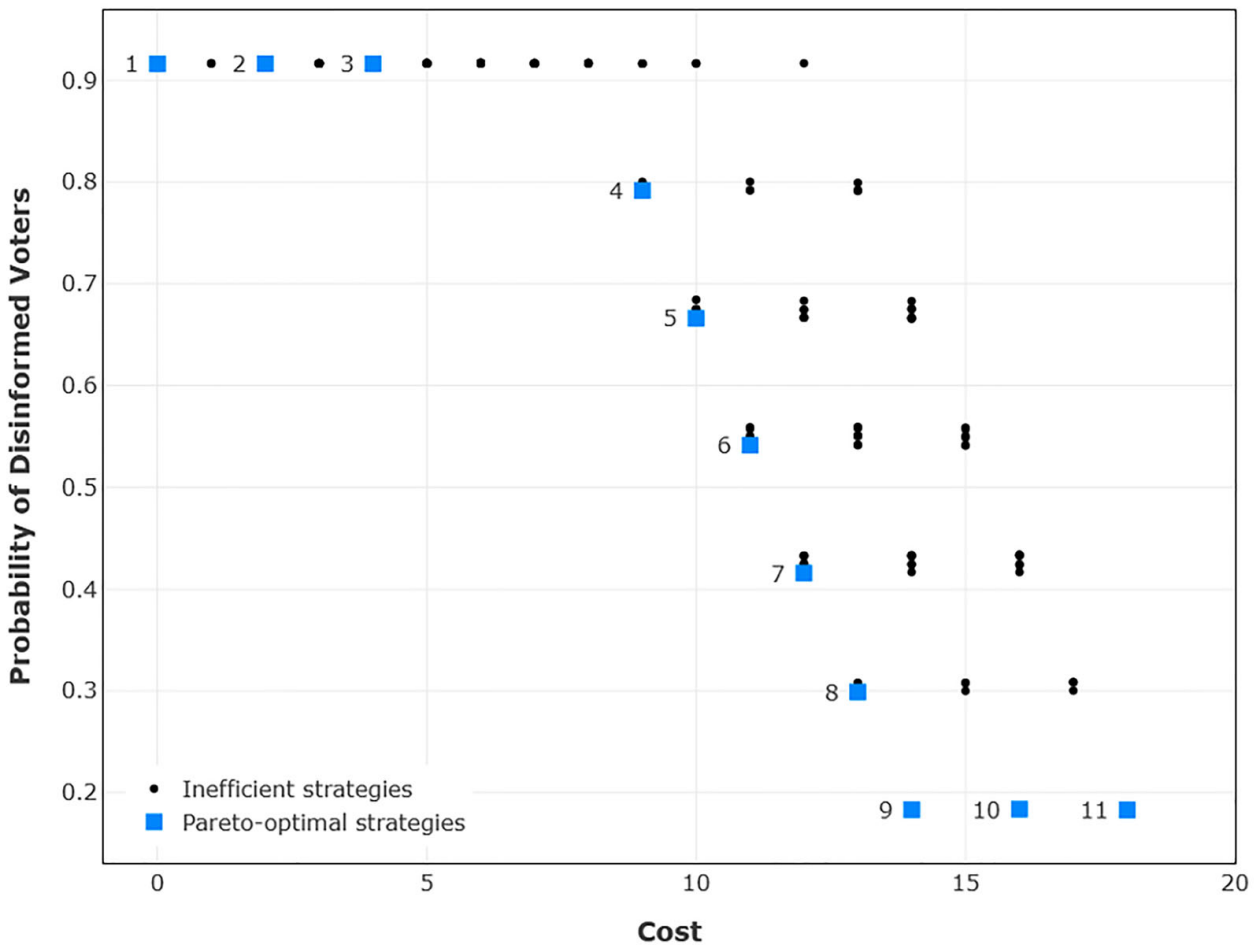
Costs and probabilities of disinformed voters for all strategies. The labels correspond to the rows in Table 1.

**FIGURE 6:**
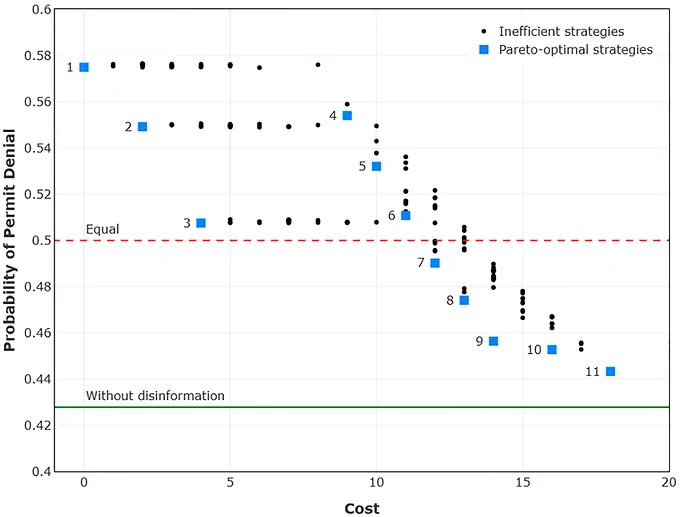
Costs and probabilities of permit denial for all strategies. The labels correspond to the rows in Table 1.

Figures 5 and 6 show the trade‐offs between the costs of risk management actions, the probability of disinformed voters, and permit denial. For the strategies above the red dashed line at 0.5 in Figure 6, permit denial is likely. The four strategies #2, #3, #10, and #11 and the three strategies #4, #5, and #6 are Pareto‐optimal, but they do not appear on the Pareto fronts in Figures 5 and 6, respectively, because these figures show only two of the three objectives (recall that Pareto‐optimality is defined with respect to *all* three objectives). Furthermore, some Pareto‐optimal strategies are unsupported, as they are dominated by linear combinations of other strategies. This is the case, for example, for Strategy #7, which lies in Figures 5 and 6 above the line segments connecting Strategies #3 and #9.

The composition of Pareto‐optimal strategies and their consequences (in boldface) are shown in Table 1. Analyzing the composition of Pareto‐optimal strategies can yield further insights. For example, strategies that cost at most 4 units consist solely of educational actions and will not reduce the probability of permit denial below 0.5. In Figure 5, the many dots to the right of Strategies #1, #2, and #3 include actions in which platforms are monitored to varying degrees of effectiveness without isolating them, but this enhanced monitoring as such does not lower the probability of permit denial. This illustrates that consequences must be analyzed systemically because the impacts of additional investments in some risk management actions depend on what other actions are implemented.

**TABLE 1:** Pareto‐optimal strategies, actions contained in them, costs, probabilities of disinformed voters and permit denial, scores (in parentheses), and overall values.

#	Monitoring			Isolation	Education	Cost of strategy	Probability of disinformed voters	Probability of permit denial	Overall value (equal weights)
	P1	P2	P3						
1	0	0	0	0	0	**0** (1.000)	**0.916** (0.000)	**0.575** (0.000)	0.333
2	0	0	0	0	1	**2** (0.943)	**0.916** (0.000)	**0.549** (0.023)	0.322
3	0	0	0	0	2	**4** (0.882)	**0.916** (0.000)	**0.507** (0.211)	0.365
4	1	0	0	1	0	**9** (0.707)	**0.792** (0.170)	**0.554** (0.014)	0.297
5	2	0	0	1	0	**10** (0.667)	**0.666** (0.341)	**0.532** (0.074)	0.361
6	2	0	1	1	0	**11** (0.624)	**0.541** (0.512)	**0.511** (0.189)	0.441
7	2	0	2	1	0	**12** (0.577)	**0.416** (0.682)	**0.490** (0.359)	0.540
8	2	1	2	1	0	**13** (0.527)	**0.299** (0.842)	**0.474** (0.538)	0.636
9	2	2	2	1	0	**14** (0.471)	**0.183** (1.000)	**0.456** (0.784)	0.752
10	2	2	2	1	1	**16** (0.333)	**0.183** (1.000)	**0.453** (0.841)	0.724
11	2	2	2	1	2	**18** (0.000)	**0.183** (1.000)	**0.443** (1.000)	0.667

To reduce the probability of a permit denial below 0.5, a combination of monitoring and isolation actions must be deployed, and the attacked platform must be isolated. Across Pareto‐optimal strategies #7 through #11, Platforms 1 and 3 should always be monitored closely (at Level 2), but Platform 2 may be monitored at any one of the three possible levels. In addition, because disinformation from Platforms 1 and 3 can reach all voters, monitoring them together is more effective than monitoring either of the other two platform pairs (Platforms 1 and 2, Platforms 2 and 3). Even if all risk management actions are implemented, at a total cost of 18 units, the probability of permit denial will not be reduced to the baseline level of 0.428 = 1 − 0.572 (where 0.572 is the initial probability of granting the permit, implied by the distribution in Figure 4). This is because monitoring does not give a fully reliable indication of the disinformation attack.

Isolation actions are essential for reducing the probability that voters become disinformed. This is because education only affects the impact of disinformation, not its propagation. For example, consider Strategies #3 and #4. Strategy #3, which consists of education actions, outperforms Strategy #4, as it has a lower probability of permit denial. Strategy #4, which consists of monitoring and isolation actions, has a lower probability of disinformed voters because it helps prevent the propagation of disinformation from the attacked platform. However, because monitoring does not provide a fully reliable indication of the disinformation attack on one platform, the probability of disinformed voters cannot be reduced below 0.183, compared with the initial probability of 0.916 when no isolation actions are implemented.

The information in Table 1 can be further synthesized with the help of value functions, defined on the range of impacts, and the criteria weights in Equation (1). For illustration, assume that the functions are power functions $v_{i}\left(x_{i}\right)={x_{i}}^{r_{i}}$, where $r_{i}>0$ is the exponent and $x_{i}\in \left[0,1\right]$ are the normalized performance scores obtained through a linear mapping of the worst and best levels of the objectives to 0 and 1, respectively. Here, the worst and best levels can be readily defined for each objective from the Pareto‐optimal strategies. Thus, for Strategy #11, whose cost is 18, the normalized performance score is 0, and 1 for the “do nothing” strategy 1, which does not cost anything. Furthermore, assume that the single‐attribute value score associated with cost level 10 is 0.667 (representing a preference for low costs, as two‐thirds of the maximum value on the cost criterion is gained by limiting costs to 10) and that the single‐attribute value score associated with the probability of disinformed voters is linear. Finally, the single‐attribute score associated with a probability of 0.5 of permit denial is 0.2 (representing a preference for small changes in election results, given that only a fifth of the maximum value on this criterion is gained by lowering it from 0.567 to 0.5). Thus, the respective exponents are $r_{1}=0.5$, $r_{2}=1$, and $r_{3}\approx 2.32$. These assumptions imply the scores in parentheses in Columns 7, 8, and 9 of Table 1.

Furthermore, if the criteria have equal weights in the additive value function representation in Equation (1), meaning that lowering costs from 18 to 0 contributes as much to the overall value as reducing the probability of disinformed voters from 0.916 to 0.183 and reducing the probability of permit denial from 0.567 to 0.442, the scores can be combined to obtain the overall values in the last column in Table 1. In this case, Strategy #9, which consists of close monitoring of all platforms and platform isolation without providing education, would have the highest overall value and be the recommended strategy.

The model can also be used to conduct sensitivity analyses on how changes in risk management actions would affect strategy recommendations. For example, assume that the effectiveness of the more substantial education program can be improved so that, for voters exposed to disinformation, the ratio between the probabilities of voting in support of granting the permit versus voting against the permit is reduced only by 20% instead of 50% (i.e., the parameter Δ(2) in Appendix A changes from 1/2 to 4/5). Then, such education *alone* would lower the probability of permit denial to 0.454, which is better than 0.456 for Strategy #9. However, the overall value function with equal criterion weights would still recommend Strategy #9 because education does not limit the spread of disinformation, and Strategy #9 has the lowest probability of disinformed voters. However, if the weight of the probability of disinformed voters was less than 0.243 (with the remaining weight divided equally between the other two criteria), improved substantial education would become the recommended strategy.

More generally, one could also explore the impacts of actions that weaken the communication connections shown in Figure 3. Platform isolation is representative of such an action, as it severs the connection between the attacked platform and its voters. For example, one could assess the impacts of weakening but not fully cutting communication connections between voters. The attractiveness of doing so would need to be evaluated by accounting for the full set of possible actions (and their costs) from which strategies can be built.

Methodologically, the novelty of this case study lies in its span of different types of risk management actions, including those focused on specific stages of the process (such as monitoring), localized interventions (such as platform isolation), and broader cross‐cutting impacts (such as education). This enables more systematic analyses and can also support the development of additional risk management actions that, when added to those previously introduced, would complement them effectively. From this perspective, the proposed approach of multi‐criteria portfolio decision analysis contributes to two of the four future research directions identified in Hunt et al. (2020), as it helps inform investment planning in mitigating mis/disinformation and makes it possible to optimize strategies for scenarios that reflect such propagation.

In the case study above, the focus was on the impacts of disinformation on a collective vote to grant a permit for a physical production facility. However, because the proposed modeling approach can (i) track the disinformation status of all information users and (ii) characterize how this status affects their behavior, it can be extended to situations where users make other kinds of decisions, such as which transportation routes they take and how much critical resources they consume. This makes it possible to link the information model with models representing the physical infrastructures affected by users' decisions. Thus, the consequences layer in Figure 2 effectively has two levels: the information level and the physical level (see Barker et al. 2025). Hence, objectives concerning the performance of the physical level (e.g., maximizing reliability at different locations over time) can be examined to develop corresponding criteria for multi‐criteria models. In addition, criteria from the information level can also be introduced (e.g., minimizing user exposure to disinformation). The resulting multi‐criteria models can then be leveraged in much the same way as in the case study above to identify strategies implemented through portfolios of risk management actions at the information level that offer the most value in terms of contributing to achieving overall objectives.

## 6 Discussion

The case study above suggests that countering the risks of mis/disinformation can benefit from a systemic approach that supports coordinated strategy decisions. Specifically, rather than making choices among “single” actions independently—such as deciding how to monitor a given platform—it is instructive to consider portfolios of complementary risk management actions and to use modeling to account for interdependencies in their impacts. Here, multi‐criteria portfolio decision analysis (see, e.g., Salo et al. 2011; Liesiö et al. 2021) helps identify Pareto‐optimal strategies from the potentially very large number of alternative strategies that can be constructed by combining distinct risk management actions. For example, Żebrowski et al. (2022) present an instructive case study on selecting portfolios of risk management actions against cyberattacks. To accommodate uncertainties in assessing the consequences of the strategies, one can explore ranges of parameter values instead of numerical point estimates, as in our case study (see, e.g., Liesiö and Salo 2012).

In the case study above, the scenario was that only one platform would be attacked. One could develop many other scenarios depicting alternative futures to identify which strategies perform particularly well in one or more of them. However, given the large number of possible scenarios, this creates a need to choose which scenarios to select (Haimes et al. 2002). Such choices can be assisted by multi‐criteria methods based on evaluation criteria such as diversity and consistency (Seeve and Vilkkumaa 2022). One can even introduce scenario probabilities that depend on the actions taken (Vilkkumaa et al. 2026). In contrast, comprehensiveness, referring to the systematic exploration of *all* possible combinations of realizations of relevant uncertainties (see, e.g., Tosoni et al. 2018), may be an unrealistic aim, as there are extraordinarily many ways in which mis/disinformation can be propagated and used in society.

While our numerical example has many parameters, these could nevertheless be estimated from data analysis (see Jiang et al. 2021; Hunt et al. 2020, 2022 for examples of extensive empirical studies) or through structured expert judgment interviews with specialists, decision makers, or stakeholders (Cooke 1991). However, the temptation to build increasingly complex models merely for the sake of analytical sophistication should be resisted. Feedback from users, especially through longitudinal case studies (see, e.g., Mild et al. 2015), can help understand how models can be best embedded in decision processes.

In our literature review, we encountered many applications of multi‐criteria methods that have paid little attention to rigor and defensibility or, more formally, the requirements for prescriptive support for rational decision‐making (see, e.g., Abrar et al. 2023; Bajpai and Chaturvedi 2024). One of these is the alignment with decision‐theoretic axioms of rationality, such as the transitivity of preferences,^3^ while the exclusion of rank reversals is another requirement for rationality.^4^ Methods that violate such requirements and axioms, such as the AHP and DEMATEL, may offer benefits, as applying any structured approach may be better than using none. Nevertheless, one can make a strong case for methods with a solid axiomatic foundation, most notably MAVT and multi‐attribute utility theory (MAUT) (see, e.g., French 1993). These methods are appealing in that the numerical parameters of decision models, such as alternative assessment scores and criterion weights, have well‐defined meanings.

## 7 Conclusion

Mis/disinformation poses complex, systemic risks that weaken the foundations of evidence‐based decision‐making and democratic governance. While traditional risk management approaches, which proceed from the identification of failure scenarios to the estimation of scenario probabilities and the assessment of consequences (see, e.g., Kaplan and Garrick 1981), can be applied to study these risks, mis/disinformation involves unique challenges that arise from the recursive and actor‐dependent communication dynamics. For example, it may be difficult to obtain reliable numerical parameter estimates for the behavior of adversaries who act surreptitiously. In addition, the societal consequences of the propagation of unprecedented false information are harder to predict than those of previously encountered physical hazards that evolve following well‐known laws of physics.

Another differentiating feature is that, from the viewpoint of upholding democratic governance and social cohesion (cf. Bennett and Livingston 2018), mis/disinformation can pose particularly pervasive and subtle threats because those in power may seek to produce and propagate it. In effect, problems arising from mis/disinformation cannot be fully isolated and addressed as an external threat because they are part of its communication fabric. Here, the close attention that the Finnish Government's Report (Government of Finland 2025) gives to media literacy, digital citizenship, and broader awareness through educational efforts is pertinent (cf. Carmi et al. 2020). Unless mis/disinformation is perceived to pose risks to society and its administrative apparatus, the associated risks are likely to persist and intensify. In the face of this reality, complacency may be the greatest risk of all.

From the perspective of policy processes, multi‐criteria methods can be advocated on the grounds that they improve the traceability of decision processes. This can be valuable as the distinction between the “external” risks (caused by natural phenomena such as extreme weather events) and the “internal” administrative apparatus (responsible for countering risks in accordance with approved standards and ethical norms) is not necessarily clear in cases of mis/disinformation, especially in the presence of political forces that may seek to disrupt legitimate governance processes. In effect, this apparatus itself may be permeated with mis/disinformation and take part in its propagation, even to the extent that the trustworthiness of administrative processes will be severely compromised.

Against this backdrop, there is a growing need for analytical rigor, methodological transparency, and stakeholder participation, which are all hallmarks of well‐designed and well‐executed decision processes. Beyond their instrumental role in providing structured multi‐criteria decision support, such processes promote accountability in risk governance by clarifying links among modeling assumptions, resulting decision recommendations, and actual decisions. This may reduce the risk that decisions will be reached on shallow or even arbitrary grounds. Here, methods of multi‐criteria portfolio decision analysis can play a role in making these processes more transparent, inclusive, and reflective. Thanks to their ability to incorporate a broad range of criteria—spanning technical and social considerations—these methods are well positioned to support the formulation of balanced policy portfolios that go beyond choices among individual risk management options. Inspired by this recognition, we see the need for further methodological and applied research that seeks to integrate these methods with other analytical approaches.

## Conflicts of Interest

The authors declare no conflicts of interest.

## Endnotes

1 These included fields such as epidemiology, psychology, economic policy, and environmental science. Resilience as a field was represented by the first author.

2 The scenario analysis was carried out in the research project “Adversarial Risk Analysis for Impact Assessment,” funded by the Scientific Advisory Board for Defence in Finland (see Roponen 2023).

3 If Alternative A is preferred to Alternative B, which is preferred to Alternative C, then it logically follows that Alternative A should be preferred to Alternative C.

4 A rank reversal occurs, for example, when the preference ranking of two alternatives changes as a result of adding the possibility to choose a third alternative which is irrelevant in the sense that it is less preferred than either one of the two alternatives being considered (see, e.g., Salo and Hämäläinen 1997; Maleki and Zahir 2013).

## Appendix A

### A.1

The network in Figure 3 representing possible information flows is modeled as a directed graph G=(V,E), where nodes $V=V^{P}\cup V^{V}$ correspond to platforms $V^{P}=\left\{P1,P2,P3\right\}$ and voters $V^{V}=\left\{A,B,C,\cdots ,Q\right\}$, connected by edges $E\subseteq \{\left(v,\;v^{\prime }\right)\;|\;v,\;v^{\prime }\in V\}$. In the absence of disinformation, the probability that voter $v\in V^{V}$ votes to grant the permit, denoted by D(v)=1, is beta‐distributed according to $p_{v}∼B\left(\alpha ,\;\beta \right)\in \left[0,\;1\right]$ (see, e.g., Rigdon et al. 2009), where α=6 and β=5.5 are the distribution parameters. Voting for permit denial is denoted by D(v)=0. For these parameters, the permit is initially granted with a probability of 0.572 and denied with a probability of 0.428.

The disinformation status is represented by a binary variable, such that $S\left(v\right)=1,\;v\in V^{P}\cup V^{V},$ if the platform $v\in V^{P}$ is attacked with disinformation or the voter $V^{V}$ becomes disinformed. In our scenario, all platforms are equally likely to be attacked $P\;\left[S(v)=1\right]=\frac{1}{3},\;\forall v\in V^{P}$. We assume there is no recovery from disinformation (see, e.g., Rocco et al. 2025).

The policymaker can take action to monitor each platform at three different levels of intensity, represented by the decision variable $\lambda_{v}^{M}$, whose value is 0, 1, or 2 in the case of no monitoring, light monitoring, and close monitoring; these three values are also the costs of the respective monitoring actions. For the monitoring action $\lambda_{v}^{M}$, the policymaker obtains a binary indication of the attack $S\left(v\right)=1,\;v\in V^{P}$ with probability $P\;\left[S^{M}\left(v\right)=1|\;S\left(v\right)=1\right]=\lambda_{v}^{M}\delta^{M}$, where $\delta^{M}=0.4$. Thus, for close monitoring $\lambda_{v}^{M}=2$, there is still a 1 − 2 × 0.4 = 0.20 probability of not detecting the disinformation. There are no false positive indications: if the indication is received, the attack has surely occurred.

If the monitoring indication is received, preparations for isolating the attacked platform $v\in V^{P}$ can be made by the action represented by the binary decision variable $\lambda^{I}=1$ at a cost of 8 units. If isolated, the disinformation cannot propagate from the platform to any voters. Because only one platform is attacked, there is no need to introduce separate decision variables for the different platforms.

If platform $v^{\prime }\in V^{P}$ is attacked but not isolated, voter $v\in V^{V}$, who is directly connected only to this platform, becomes disinformed with probability $q_{v^{\prime },v\;}=\delta^{P}$, where parameter $\delta^{P}=0.5$ represents the strength of this connection. Disinformation can propagate from disinformed voters to their respective connections so that if voter $v^{\prime }\in V^{V}$ is disinformed $S(v^{\prime })=1$, then the influence of this on voter $v\in V^{V}$, connected to $v^{\prime }$ by edge $(v^{\prime },\;v)\in E,$, is $q_{v^{\prime },v\;}=\left(1-p_{v^{\prime },v\;}\right)\delta^{V}$, where parameter $\delta^{V}=0.4$ represents the strength of the connection between voters. This equation reflects the reality that supporters of granting the permit, who have a high value of $p_{v^{\prime }}$, are less likely to promote disinformation in support of permit denial. Finally, a voter $v\in V^{V}$ with incoming connections from disinformed nodes $V^{D}\left(v\right)=\{v^{\prime }\in V\;|\;\left(v^{\prime },\;v\right)\in E,\;S\;\left(v^{\prime }\right)=1\}$ becomes disinformed with probability

which is the noisy OR‐gate model (Oniśko et al. 2001). The disinformation status of voters is simulated sequentially in the order given in Figure 3.

Disinformation influences voters' behavior by affecting the likelihood ratio representing their probabilities of voting to grant or deny. Specifically, for a disinformed voter $v\in V^{V},\;S\;\left(v\right)=1$, this ratio changes from $p_{v}/\left[1-p_{v}\right]$ to $\frac{q_{v}}{1-q_{v}}=\Delta \left(\lambda^{E}\right)\frac{p_{v}}{1-p_{v}},$where $q_{v}$ the updated probability of granting the permit and the parameter $\Delta (\lambda^{E})$ reflects the impact that the decision $\lambda^{E}$ to educate voters has on the impact of disinformation. Education can be provided at three levels: $\lambda^{E}=0$ for no education, $\lambda^{E}=1$ for limited education, and $\lambda^{E}=2$ for substantial education. The associated costs are twice these values. Parameters $\Delta (\lambda^{E})$ are assumed to be Δ(0)=1/4,Δ(1)=1/3,Δ(2)=1/2, which yields the corresponding distributions shown in Figure 4. For example, if the initial probability of voting to grant is $p_{v}=3/4=0.75$ and no education is provided, then $\lambda^{E}=0$ and $\Delta (\lambda^{E}=1/4)$, in which case the probability of voting to grant is reduced to $q_{v}=3/7=0.423$.

The possible strategies are portfolios $\lambda =(\lambda_{P1}^{M},\lambda_{P2}^{M},\lambda_{P3}^{M},\lambda^{I},\lambda^{E})\in \Lambda$ defined by combinations of monitoring, isolation, and education actions. The first objective is to minimize the total cost of actions. Second, the probability of disinformed voters is minimized. Third, the impact on the outcome of the vote is to be minimized, which is interpreted as minimizing the probability that the permit is denied, further to the disinformation attack. Thus, the multi‐criteria optimization problem is

$$\underset{\lambda \in \Lambda }{v-\min}\;\left(\begin{array}{c} \;\sum_{v\in V^{P}}\left(c^{M}\lambda_{v}^{M}\right)+c^{I}\lambda^{I}+c^{E}\lambda^{E},\\ \;P\left[\sum_{v\in V^{V}}S\left(v\right)>0\;|\;\lambda \right],\\ \;P\left[\sum_{v\in V^{V}}D\left(v\right)<\frac{\left|V^{V}\right|}{2}\;|\;\lambda \right] \end{array}\right),$$

where $c^{M},\;c^{I},\;c^{E}$ are the costs associated with monitoring, isolation, and education, respectively, and $|V^{V}|$ is the number of voters.
